# Structural Determination of a Human IgE Epitope on Major Birch Allergen Bet v 1

**DOI:** 10.1111/all.70240

**Published:** 2026-02-01

**Authors:** Andrea O'Malley, Brooke T. Borders, Jeffrey M. Wilson, Scott A. Smith, Maksymilian Chruszcz

**Affiliations:** ^1^ Department of Biochemistry and Molecular Biology Michigan State University East Lansing Michigan USA; ^2^ Department of Pathology, Microbiology and Immunology Vanderbilt University Medical Center Nashville Tennessee USA; ^3^ Division of Asthma, Allergy, and Clinical Immunology University of Virginia School of Medicine Charlottesville Virginia USA

**Keywords:** allergens and epitopes, IgE, pollen

**Abbreviations**
CDR: complementary determining region
Fab: antigen binding fragment
hIgE: human immunoglobulin E
mAb: monoclonal antibody
mIgG: murine immunoglobulin G
PR‐10: pathogenesis‐related group 10

To the Editor,

PR‐10 allergen Bet v 1 (*Betula pendula*, white birch) is considered one of the most important allergens in pollen allergy, with up to 95% of birch‐allergic individuals having IgE against the protein [1, 2]. Furthermore, Bet v 1 is implicated in pollen‐food allergy syndrome, which occurs when a Bet v 1‐sensitized individual experiences oral‐adjacent symptoms in response to eating raw fruits, vegetables, or nuts; this is caused by IgE‐mediated cross‐reactivity among PR‐10 allergens [3]. The IgG antibody repertoire binding Bet v 1 has been previously described for four structures of Bet v 1 bound to murine IgG (mIgG) antibody fragments (Fabs) or humanized antibodies [4, 5]; however, no structures have been described for human IgE (hIgE) Fabs bound to PR‐10 allergens.

Use of hIgE monoclonal antibodies (hIgE mAbs) isolated from human B cells allow for the physiological examination of the allergen‐antibody response [6]. Therefore, we pursued the isolation of hIgE mAbs from PR‐10‐allergic patients to evaluate IgE epitopes on the surface of Bet v 1. hIgE mAb 2H22 was shown using ELISA EC_50_ assays with recombinant protein to bind PR‐10 allergens, including Bet v 1.0101, Fag s 1.0101, Fra a 1.0102, Pru p 1.0101, and Que a 1.0301 (Table S1 and Figure S1).

Here, we describe the crystal structure of an Fab derived from hIgE mAb 2H22 bound to Bet v 1.0101, structurally demonstrating the first hIgE epitope on a PR‐10 allergen. The 2H22 epitope centers on the C‐terminal side of Bet v 1.0101, with the majority of the interactions on the loop between β‐strands β6 and β7 (Figure 1A). PDBePISA measures the epitope interface to 822 Å^2^, with interactions generated with all three CDRs of the heavy chain and CDR1 and CDR2 of the light chain. The heavy chain forms hydrogen bonds with Glu9, Ala107, Thr108, and Pro109 of Bet v 1.0101, and the light chain generates bonds with Glu9, Asp110, and Lys116 (Figure 1B). The most significant interactions of Bet v 1 with the antibody are formed by hydrophobic interactions of Pro109 with Trp110 of the heavy chain (CDR3) and similar close contacts with Tyr50 (adjacent to CDR2) of the light chain. Interestingly, the epitope of hIgE mAb 2H22 overlaps slightly with the epitope for IgG REGN5714 (PDB: 7MXL) (Figure 1C).

**FIGURE 1:**
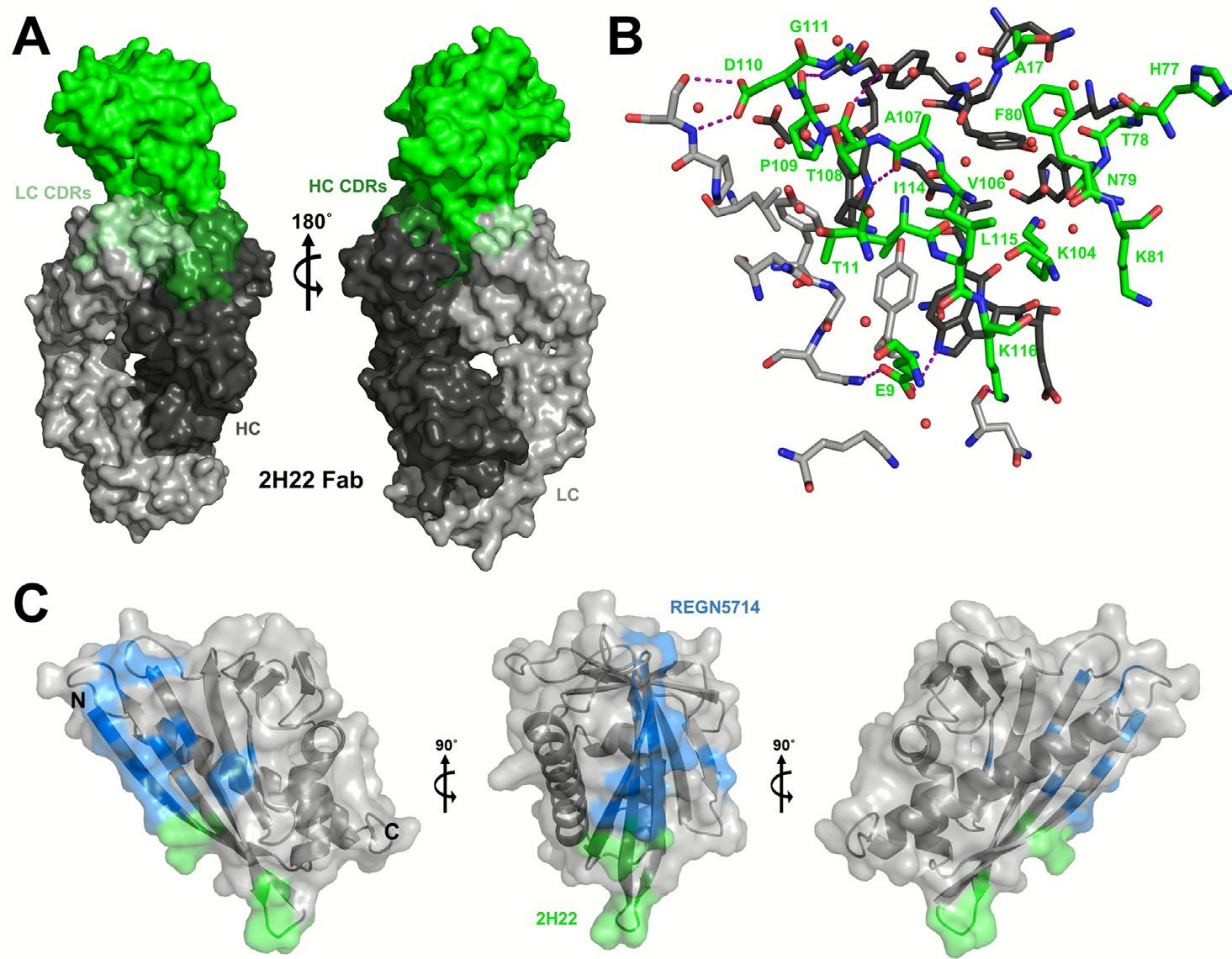
Structural evaluation of the hIgE 2H22 epitope on Bet v 1 (green). (A) Overview of the hIgE Fab 22H2‐Bet v 1.0101 complex. The heavy chain is colored in dark gray, the light chain in light gray, the CDRs for the heavy chain in dark green, and the CDRs for the light chain in pale green. (B) hIgE 22H2 epitope on Bet v 1.0101. Hydrogen bonds are shown as purple dashes and water molecules in the allergen‐antibody interface are shown as red spheres. Residues from the heavy chain are shown in dark gray, and residues from the light chain are colored in light gray. (C) Comparison of the REGN5714 epitope (blue) to that of 2H22 (green) on Bet v 1 (gray). Residues forming hydrogen bonds with the respective antibodies are colored. The N‐ and C‐terminals of Bet v 1 are labeled accordingly.

Comparison of the 2H22‐Bet v 1 complex to the previously published IgG Fab‐Bet v 1 complexes demonstrates a novel epitope (Figure 2). The 2H22 epitope overlaps slightly with the epitope for REGN5714, is next closest to REGN5713, and is on the opposite side of the protein for the binding sites of BV16 and REGN5715. While none of the Bet v 1 residues involved in the 2H22 or REGN5714 epitopes form hydrogen bonds or salt bridges with both antibodies, several common residues are buried within the interface for each epitope, suggesting slight overlapping (Table S2). Further experiments would be required to determine if the binding of 2H22 to Bet v 1 would inhibit the interaction of Bet v 1 with any of the other determined antibodies.

**FIGURE 2:**
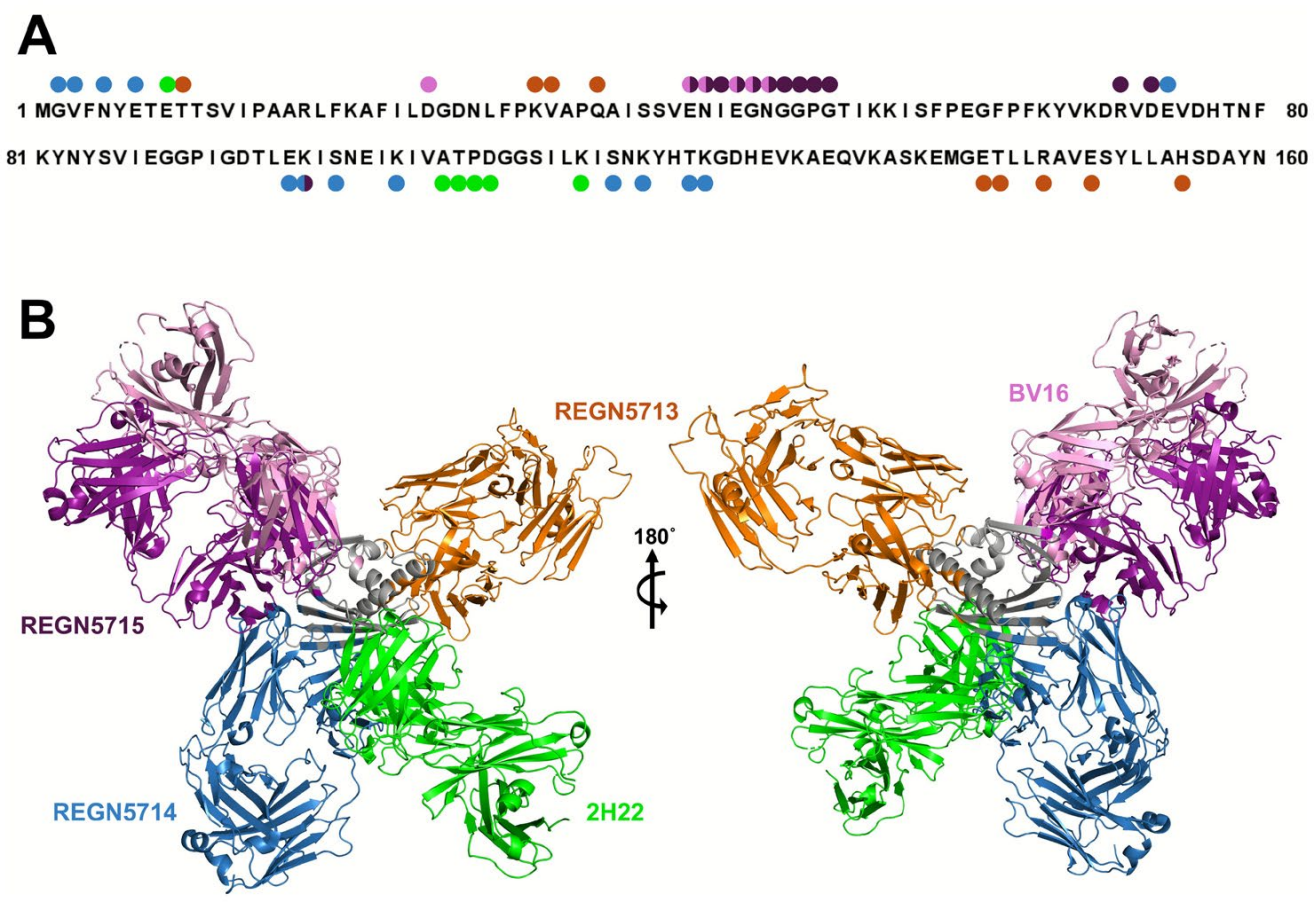
Comparison of the hIgE 2H22 epitope on Bet v 1 with those of previously published IgG mAbs. (A) Residues on Bet v 1.0101 making hydrogen bonds or salt bridges with Fabs, as calculated by PDBePISA. Residues for hIgE Fab 2H22 are marked in green, IgG Fab REGN5713 in orange, mIgG Fab REGN5714 in blue, IgG Fab REGN5715 in pink, and mIgG Fab BV16 in purple. (B) Superimposed structures of Fabs binding Bet v 1 (gray). Colors of Fabs are as described for (A). Residues making hydrogen bonds or salt bridges are colored accordingly on Bet v 1.

In summary, the crystal structure of hIgE Fab 2H22 in complex with Bet v 1 shows the first IgE epitope on a major PR‐10 allergen. The 2H22 epitope represents a novel epitope on the surface of Bet v 1, suggesting that there are more IgE binding sites on Bet v 1 that have not yet been elucidated. This structural data can be used for the proposal of hypoallergens or other allergy treatment methods.

## Author Contributions

All authors contributed according to Allergy's authorship criteria, including involvement in the study's design, analysis, or manuscript revision, and have approved the final version of the manuscript.

## Funding

This work was supported by a research grant from NIH/National Institute of Allergy and Infectious Disease R01AI155668 (to S.A.S.). This research used resources of the Advanced Photon Source, a U.S. Department of Energy (DOE) Office of Science User Facility operated for the DOE Office of Science by Argonne National Laboratory under Contract No. DE‐AC02‐06CH11357. Use of the LS‐CAT Sector 21 was supported by the Michigan Economic Development Corporation and the Michigan Technology Tri‐Corridor (Grant 085P1000817). This work was also supported by startup funds from Michigan State University.

## Ethics Statement

The studies involving humans were approved by Vanderbilt University Medical Center Institutional Review Board (IRB 141330 and 142030). The studies were conducted in accordance with the local legislation and institutional requirements. Written informed consent was obtained from all subjects prior to enrollment in the study.

## Conflicts of Interest

S.A.S. receives royalties for intellectual property licenses with InBio and consulting fees from IgGenix Inc. He is an inventor on a patent entitled “Generation of human allergen‐ and helminth‐specific IgE monoclonal antibodies for diagnostic and therapeutic use” (US patent no. US10908168‐B2), with royalties paid, and on a pending patent entitled “Generation of human peanut allergen‐specific IgE monoclonal antibodies for diagnostic and therapeutic use” (63/159,764) with royalties paid. The other authors declare no conflicts of interest.

## Supporting information


**Data S1:** all70240‐sup‐0001‐Supinfo.docx.

## Data Availability

Structures described in this work have been deposited in the PDB with codes 9Y0A, 9Y0D, and 9Y0E. Diffraction images are available from the authors upon request.
