# Supplementary material for: Structural Determination of a Human IgE Epitope on Major Birch Allergen Bet v 1
Source: Allergy. 2026 Feb 1;81(6):2201–3. doi: 10.1111/all.70240 (PMC13256281; doi:10.1111/all.70240)
Supplement: Supplementary file 1 — Data S1: all70240‐sup‐0001‐Supinfo.docx. [file ALL-81-2201-s001.docx]

**Structural determination of a human IgE epitope on major birch allergen Bet v 1: supplemental information**

**Supplemental Results**

*Specificity assays*

The ALEX_2_ (Macro Array Diagnostics) macroarray test was used to confirm the binding of hIgE mAb 2H22 to PR-10 allergens, demonstrating some level of binding to six PR-10 allergens as well as one extract and one allergen mixture. ELISA EC_50_ assays determined that IgE mAb 2H22 bound recombinant Pru p 1.0101 (*Prunus persica*, peach), Que a 1.0301 (*Quercus alba*, oak), Bet v 1.0101, and Fra a 1.0102 (*Fragaria ananassa*, strawberry) with highest affinity respectively. 95% confidence intervals for each allergen confirmed good binding for each. Figure S1 shows EC_50_ plots for selected PR-10s, and Table S1 shows all EC_50_ values for PR-10s produced in this work.

*Additional crystal structures*

The 2H22 Fab-Bet v 1 complex also crystallized in the *C*2 space group. This crystal diffracted to 2.90 Å, and the structure also has one complex in the asymmetric unit. The two structures superimpose with an RMSD of 0.6 Å (over 538 Cα atoms), demonstrating very few conformational changes between the structures (Figure S2A). The primary variations are in the sugar moiety linked to Asn17 of the light chain. As with the complex described in the main text, the hydrophobic cavity of Bet v 1.0101 contains two polyethylene glycol molecules from the crystallization condition, which is consistent with PR-10 allergens binding small ligands^1^. There are some changes in the Bet v 1 loops between this structure and the higher resolution structure (Figure S2B); both of these differences are consistent with overall protein flexibility and changes in the crystallization condition.

The 2H22 Fab crystallized in the *P*6_5_ space group and diffracted to 2.27 Å. All residues could be modeled except for Lys140, Ser141, and Thr142 in the heavy chain. The Fab alone superimposes to the complexes with an RMSD of 1.3-1.6 Å (over 410-422 Cα atoms), demonstrating that the CDRs did not significantly move in the absence of the allergen (Figure S2C). Furthermore, the sugar moiety is comparable to that observed in the complexes.

*Further cross-reactivity of hIgE Fab 2H22*

We tested a panel of 16 PR-10 allergens from pollen, fruit, and nuts with hIgE mAb 2H22 to evaluate the allergens’ cross-reactivity (Table S1). The lowest EC_50_ was found for Pru p 1.0101, Que a 1.0301, Bet v 1.0101, Fra a 1.0102, and Fag s 1.0101 (*Fagus sylvatica*, European beech). Since, of the pollen allergens, the lowest EC_50_ was for Que a 1.0301, hIgE mAb 2H22 may be specific to Que a 1.0301.

The tested PR-10 allergens have 38-81% sequence identity to Bet v 1.0101 (Figure S3). Sequence comparison of the allergens shows that the primary residues that form the hIgE mAb 2H22 epitope, Glu9, Ala107, Thr108, Pro109, Asp110, and Lys116, are not conserved across all tested PR-10s. The variation in these residues, primarily the loop formed by Ala107-Asp110, provides preliminary explanation for the variation in binding for the mAb. The 27 Bet v 1 isoforms that are registered by the WHO/IUIS Allergen Nomenclature Subcommittee^2^ do not have significant sequence variation in the residues that form the epitope (data not shown), suggesting that any other Bet v 1 isoallergen should also bind hIgE mAb 2H22.

**Supplemental Materials and Methods**

The studies involving humans were approved by Vanderbilt University Medical Center Institutional Review Board (IRB 141330 and 142030). The studies were conducted in accordance with the local legislation and institutional requirements. Written informed consent was obtained from all subjects prior to enrollment in the study.

hIgE mAb 2H22 was isolated from serum from a patient with allergic rhinitis and food allergy, with methods previously described for 2F10 and 4C8 IgE mAbs^3,4^. The mAb was sequenced and the Fab was generated using the IgE variable regions expressed with human IgG constant domains, resulting in correctly paired light and heavy chains. The Fab was produced by GenScript in CHO cells. The Fab then was purified using Capture Select CH1-XL 5 mL chromatography column (Thermo 494346205).

DNA encoding for selected PR-10s (Table S1) were inserted into the pET-26b(+) vector by Synbio Technologies (Monmouth Junction, NJ), with sequences taken from allergen.org. DNA was transformed into DH5α *E. coli* cells, and plasmids were purified and transformed into BL-21 *E. coli* cells. 1 L LB cultures were inoculated with 10 mL LB cultures with transformed cells and kanamycin for selection, grown at 37˚C to an OD of 0.6-0.8, and induced with IPTG and grown overnight at 16˚C. Cultures were spun down and pellets were solubilized with lysis buffer (50 mM Tris-HCl pH 7.5, 500 mM NaCl, 30 mM imidazole, 2% glycerol) and sonicated. Subsequent lysate was spun down and the supernatant was purified with ammonium sulfate purification. The protein precipitated out at 80-100% ammonium sulfate, and pellets were resuspended in Tris buffered saline (TBS buffer; 50 mM Tris-HCl pH 7.5, 150 mM NaCl) before undergoing further purification with anion exchange chromatography with a salt gradient (buffer A: 50 mM Tris-HCl pH 7.5; buffer B: 50 mM Tris-HCl pH 7.5, 2 M NaCl). Finally, size exclusion chromatography in TBS buffer for crystallization experiments or phosphate buffered saline (PBS) buffer for antibody affinity assays was performed to finish purification of the PR-10s.

ALEX_2_ macroarray tests (Macro Array Diagnostics) were performed to determine the allergen family to which the mAb was generated against and provide a suggestion of specificity. ELISA assays (EC_50_) were performed to determine the specificity of the hIgE mAb based on the PR-10s generated. ELISA EC_50_ assays were performed using an Integra Assist Plus pipetting robot. Purified PR-10 allergens (in PBS buffer) were used at 10 µg/mL to coat 384-well ELISA plates. A 24-well 1:2 dilution series of purified human IgE mAb was generated, starting at a concentration of 10 mg/mL and transferred in triplicate into blocked PR-10 coated wells, or block-only wells as a negative control. Following washing, the mouse anti-human IgE FC-HRP secondary antibody will be added prior to secondary washing and adding substrate solution. Data was analyzed using GraphPad Prism, with EC_50_ values and 95% confidence intervals expressed as ng/mL.

The Bet v 1.0101-2H22 Fab complex was generated by mixing purified Fab with purified PR-10 at a 1:1 ratio and incubated on ice for 30 minutes prior to SEC with TBS buffer. Fractions containing complex were concentrated to 1-10 mg/mL and plated with commercial screens with the vapor diffusion sitting drop method in Intelli-Plate 96-2 Original and Intelli-Plate 96-3 LVR plates (Hampton Research, Aliso Viejo, CA). The higher resolution complex and the Fab alone crystallized at 4˚C, and the lower resolution complex crystallized at 25˚C. The crystallization conditions are shown in Table S3.

Data was processed with HKL-3000^5^. Molecular replacement was used with HKL-3000 and MOLREP^6^. The lower resolution complex was solved with an AlphaFold3^7^ model of the Fab alone, followed by a structure of Bet v 1 (PDB: 4A80) as a search model. This structure was used for molecular replacement for the other complex. The solved Fab was used for molecular replacement for the structure of the Fab alone. Structure refinement was performed with Coot^8^ and REFMAC^9^. The TLSMD server^10^ was used to derive TLS groups for the final stages of refinement. MolProbity^11^ and Coot were used for structure validation. Data collection and structure refinement statistics are shown in Table S3.

**Figures and Tables**


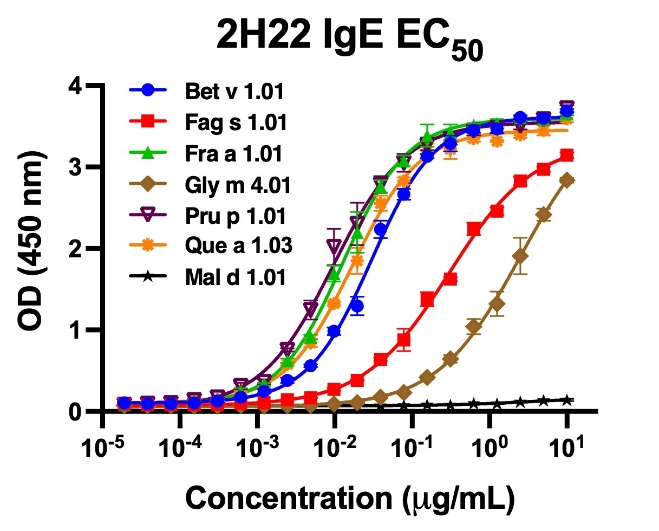


**Figure S1**. ELISA EC_50_ graphs for the interaction of hIgE mAb 2H22 with selected PR-10 allergens. Plots represent the mean ± SD of dilutions assayed in triplicate. Calculated EC_50_ values are shown in Table S1.


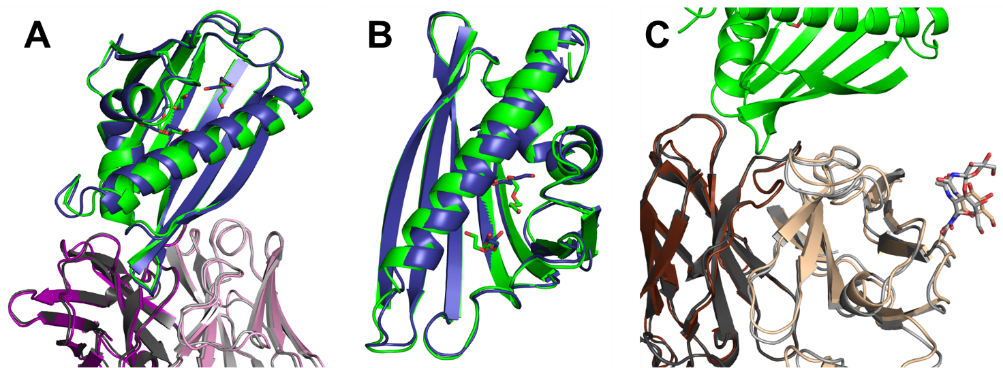


**Figure S2**. Comparison of the higher and lower resolution structures for the complex of hIgE 2H22 Fab and Bet v 1, as well as the Fab alone, demonstrating the overall conformational conservation among the three structures. The higher resolution structure is shown in green (Bet v 1), dark gray (heavy chain), and light gray (light chain), and the lower resolution structure is shown in dark blue (Bet v 1), purple (heavy chain), and pink (light chain). The Fab alone is shown in brown (heavy chain) and wheat (light chain). A) Superimposition of the complexes. B) Superimposition of Bet v 1 in the complexes, showing the variation of polyethylene glycol molecules (stick representation) in the hydrophobic cavities. C) Superimposition of the structures of the higher resolution complex with the Fab alone. The N-acetylglucosamine molecules and Asn17 are shown in stick representation for each light chain.

**
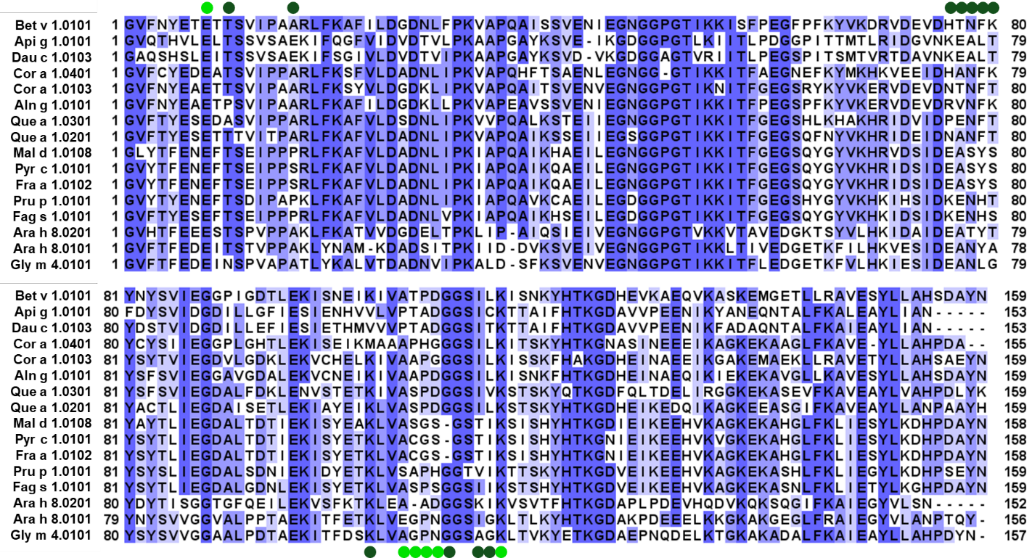
**

**Figure S3:** Sequence alignment for PR-10 allergens generated in this work. Residues that make hydrogen bonds or salt bridges with hIgE mAb 2H22 are marked with light green circles, and additional residues that make close contacts in the epitope are marked with dark green circles.

**Table S1**: EC_50_ values for the interaction of 2H22 IgE mAb with recombinant allergens produced in this work. Dashes indicate that the value was greater than 1 microgram per milliliter. CI: confidence interval.

| **Allergen** | **Source** | **Route** | **EC_50_ (ng/mL)** | **95% CI** |
| --- | --- | --- | --- | --- |
| Aln g 1.0101 | Alder | Inhalation | - | - |
| Api g 1.0101 | Celery | Ingestion | - | - |
| Ara h 8.0101 | Peanut | Ingestion | - | - |
| Ara h 8.0201 | Peanut | Ingestion | - | - |
| Bet v 1.0101 | Silver birch | Inhalation | 30 | 28-32 |
| Cor a 1.0103 | Hazel pollen | Inhalation | - | - |
| Cor a 1.0401 | Hazelnut | Ingestion | - | - |
| Dau c 1.0103 | Carrot | Ingestion | - | - |
| Fag s 1.0101 | Beech | Inhalation | 297 | 268-331 |
| Fra a 1.0102 | Strawberry | Ingestion | 40 | 33-48 |
| Gly m 4.0101 | Soybean | Ingestion | - | - |
| Mal d 1.0108 | Apple | Ingestion | - | - |
| Pru p 1.0101 | Peach | Ingestion | 10 | 9-11 |
| Pyr c 1.0101 | Pear | Ingestion | - | - |
| Que a 1.0201 | White oak | Inhalation | - | - |
| Que a 1.0301 | White oak | Inhalation | 17 | 16-18 |

**Table S2**: Comparison of Bet v 1 epitopes in published structures. Bet v 1 residues shown have at least 2 Å^2^ buried surface area within the epitope as calculated by PDBePISA^12^. Chains A (Bet v 1.0101), B (light chain), and C (heavy chain) were used for analysis for mIgG BV16. Numbering for Bet v 1.0101 residues in this structure is altered such that Gly1 in the structure is Gly2 as in the mature protein and in the other structures. Residues with asterisks are shared between the epitopes for hIgE mAb 2H22 and up to two of the mIgG epitopes. HC: heavy chain; LC: light chain.

| **hIgE 2H22**  **PDB: 9Y0A** | | **mIgG REGN5713**  **PDB: 7MXL** | | **mIgG REGN5714**  **PDB: 7MXL** | | **mIgG REGN5715**  **PDB: 7MXL** | | **mIgG BV16**  **PDB: 1FSK** | |
| --- | --- | --- | --- | --- | --- | --- | --- | --- | --- |
| **HC** | **LC** | **HC** | **LC** | **HC** | **LC** | **HC** | **LC** | **HC** | **LC** |
| Glu9*  Ala17  His77*  Thr78  Asn79  Phe80  Lys81  Lys104*  Val106*  Ala107  Thr108  Pro109  Gly111  Ile114  Leu115  Lys116* | Glu9*  Thr11*  Val106*  Thr108  Pro109  Asp110  Ile114  Lys116* | Thr11*  Ser12  Val13  Lys33  Val34  Gln37  Glu142  Thr143  Arg146  Ala147  Glu149  Ser150  Tyr151  Leu153  Ala154  His155 | Pro32  Lys33  Val34  Pro36  Gln37  Ser40  Pro60  Glu61  His155 | Gly2  Val3  Asn5  Glu7  Arg71  Asp73  Glu74  Asn83  Ser85  Glu97  Lys98  Ser100  Asn101  Glu102  Ser118  Asn119  Lys120  His122  Thr123  Lys124  Gly125 | Val3  Phe4  Asn5  Glu7  Glu9*  Asp76  Asn79  Lys81  Glu102  Lys104*  Val106*  Lys116*  Ser118 | Leu25  Asp26  Asp28  Asn29  Glu43  Asn44  Ile45  Glu46  Gly47  Asn48  Gly49  Gly50  Pro51  Gly52  Lys56  Val75  His77* | Glu43 | Glu43  Asn44  Ile45  Glu46  Gly47  Asn48  Pro51  Gly52  Thr53  Ile53  Lys69  Arg71  Asp73  Glu74  Ile87  Glu88  Lys98 | Leu25  Asn48  Gly49  Gly50  Pro51  Gly52  Asp73  Glu74  His77* |

**Table S3**: Data collection and structure refinement statistics for structures described in this work. PEG: polyethylene glycol; MME: monomethyl ether; AU: asymmetric unit; RMSD: root mean square deviation.

| **Structure** | **2H22-Bet v 1 complex** | **2H22-Bet v 1 complex** | **2H22 Fab** |
| --- | --- | --- | --- |
| PDB accession code | 9Y0A | 9Y0E | 9Y0D |
| **Data Collection** | | | |
| Crystallization conditions | 0.1 M HEPES pH 7.5  0.05 M MgCl_2_ x 6H_2_O  30% PEG MME 550 | 0.04 M KH_2_PO_4_  16% PEG 8,000  20% glycerol | 0.1 M citric acid pH 3.5  25% PEG 3,350 |
| Beamline | ESRF ID-23-2 | ESRF ID-30a | MAX-IV BioMax |
| Wavelength (Å) | 0.873 | 0.968 | 0.729 |
| **Unit Cell Parameters** | | | |
| a, b, c (Å) | 81.9, 117.5, 158.6 | 151.0, 70.3, 78.2 | 116.8, 116.8, 71.1 |
| α, β, γ (°) | 90, 90, 90 | 90.0, 116.4, 90.0 | 90.0, 90.0, 120.0 |
| Space group | *C*222_1_ | *C*2 | *P*6_5_ |
| Solvent content (%) | 57 | 58 | 60 |
| Protein chains in AU | 3 | 3 | 2 |
| Resolution range (Å) | 40.00-2.12 | 40.00-2.90 | 40.00-2.27 |
| Highest resolution shell (Å) | 2.16-2.12 | 2.95-2.90 | 2.31-2.27 |
| Unique reflections | 43,908 (2,152) | 14,960 (443) | 25,393 (1,269) |
| Redundancy | 12.6 (11.1) | 5.5 (2.0) | 11.1 (12.2) |
| Completeness (%) | 99.8 (99.7) | 91.8 (53.4) | 99.3 (100.0) |
| R_merge_ | 0.087 (0.730) | 0.100 (0.260) | 0.144 (0.911) |
| R_pim_ | 0.026 (0.224) | 0.044 (0.186) | 0.048 (0.268) |
| R_meas_ | 0.091 (0.764) | 0.110 (0.322) | 0.153 (0.951) |
| CC1/2 | 0.998 (0.866) | 0.988 (0.826) | 0.987 (0.834) |
| Average I/σ(I) | 25.5 (2.1) | 15.1 (2.2) | 25.1 (2.2) |
| **Refinement** | | | |
| R_work_ (%) | 17.3 (26.0) | 19.9 (27.2) | 18.9 (24.2) |
| R_free_ (%) | 21.8 (31.8) | 25.7 (29.7) | 24.4 (26.8) |
| Mean B value (Å^2^) | 59.2 | 52.9 | 79.4 |
| RMSD bond lengths (Å) | 0.007 | 0.005 | 0.003 |
| RMSD bond angles (°) | 1.573 | 1.245 | 0.991 |
| No. of amino acid residues | 598 | 596 | 437 |
| No. of water molecules | 372 | 51 | 167 |
| No. of other ligands | 8 | 3 | 4 |
| **Ramachandran Plot** | | | |
| Most favored regions (%) | 98.0 | 95.2 | 98.3 |
| Additional allowed regions (%) | 2.0 | 4.8 | 1.7 |

**References**

1. McBride JK, Cheng H, Maleki SJ, Hurlburt BK. Purification and Characterization of Pathogenesis Related Class 10 Panallergens. *Foods.* 2019;8(12).

2. Pomés A, Davies JM, Gadermaier G, et al. WHO/IUIS Allergen Nomenclature: Providing a common language. *Mol Immunol.* 2018;100:3-13.

3. Khatri K, Richardson CM, Glesner J, et al. Human IgE monoclonal antibody recognition of mite allergen Der p 2 defines structural basis of an epitope for IgE cross-linking and anaphylaxis. *PNAS Nexus.* 2022;1(3):pgac054.

4. Ball A, Khatri K, Glesner J, et al. Structural analysis of human IgE monoclonal antibody epitopes on dust mite allergen Der p 2. *J Allergy Clin Immunol.* 2024.

5. Minor W, Cymborowski M, Otwinowski Z, Chruszcz M. HKL-3000: the integration of data reduction and structure solution--from diffraction images to an initial model in minutes. *Acta Crystallogr D Biol Crystallogr.* 2006;62(Pt 8):859-866.

6. Vagin A, Teplyakov A. Molecular replacement with MOLREP. *Acta Crystallogr D Biol Crystallogr.* 2010;66(Pt 1):22-25.

7. Abramson J, Adler J, Dunger J, et al. Accurate structure prediction of biomolecular interactions with AlphaFold 3. *Nature.* 2024;630(8016):493-500.

8. Emsley P, Cowtan K. Coot: model-building tools for molecular graphics. *Acta Crystallogr D Biol Crystallogr.* 2004;60(Pt 12 Pt 1):2126-2132.

9. Murshudov GN, Skubák P, Lebedev AA, et al. REFMAC5 for the refinement of macromolecular crystal structures. *Acta Crystallogr D Biol Crystallogr.* 2011;67(Pt 4):355-367.

10. Painter J, Merritt EA. Optimal description of a protein structure in terms of multiple groups undergoing TLS motion. *Acta Crystallogr D Biol Crystallogr.* 2006;62(Pt 4):439-450.

11. Davis IW, Leaver-Fay A, Chen VB, et al. MolProbity: all-atom contacts and structure validation for proteins and nucleic acids. *Nucleic Acids Res.* 2007;35(Web Server issue):W375-383.

12. Krissinel E, Henrick K. Inference of macromolecular assemblies from crystalline state. *J Mol Biol.* 2007;372(3):774-797.
